# VSG-FC: A Combined Virtual Sample Generation and Feature Construction Model for Effective Prediction of Surface Roughness in Polishing Processes

**DOI:** 10.3390/mi16060622

**Published:** 2025-05-25

**Authors:** Dapeng Yang, Shenggao Ding, Lifang Pan, Yong Xu

**Affiliations:** 1 School of Computer Engineering, Jimei University, Xiamen 361021, China; yangdp1999@163.com (D.Y.); shengg@jmu.edu.cn (S.D.); 2 School of Science, Jimei University, Xiamen 361021, China; 3 Xiamen Key Laboratory of Intelligent Fishery, Xiamen Ocean Vocational College, Xiamen 361100, China

**Keywords:** surface roughness prediction, polishing, virtual sample generation, feature construction, explainability

## Abstract

Surface roughness is a critical indicator for assessing the quality and characteristics of workpieces, the accurate prediction of which can significantly enhance production efficiency and product performance. Data-driven methods are efficient ways for predicting surface roughness in polishing processes, which generally depend on large-scale samples for model training. However, obtaining an adequate amount of training data during the polishing process can be challenging due to constraints related to cost and efficiency. To address this issue, a novel surface roughness prediction model, named VSG-FC, is proposed in this paper that integrates Genetic Algorithm-driven Virtual Sample Generation (GA-VSG) and Genetic Programming-driven Feature Construction (GP-FC) to overcome data scarcity. This approach optimizes the feature space through sample augmentation and feature reconstruction, thereby enhancing model performance. The VSG-FC method proposed in this paper has been validated using data from two polishing experiments. The results demonstrate that the method offers significant advantages in both the quality of the generated virtual samples and prediction accuracy. Additionally, the proposed model is explainable and could successfully identify key influencing machining factors.

## 1. Introduction

The microscopic morphology of a workpiece surface plays a crucial role in determining its wear resistance, corrosion resistance, and tribological behavior, which affect the operational life and reliability of the product. Surface roughness is often considered a key indicator of the surface morphology and quality of a machined workpiece [1]. However, traditional roughness measurement methods often require significant time and resources in real-world manufacturing environments. As a result, they are inefficient for large-scale or high-precision production [2]. Therefore, balancing high measurement accuracy with reduced inspection time and costs remains a key challenge in advanced manufacturing [3].

With the rapid advancement of artificial intelligence technology, data-driven approaches have found successful applications across various fields. In the manufacturing industry, an increasing number of researchers are exploring AI algorithm-based data-driven methods to tackle the challenges of surface roughness prediction. Current AI-based prediction techniques are primarily divided into traditional machine learning approaches and deep learning-based [4].

Machine learning-based surface roughness modeling approaches typically use machining parameters as decision variables [5]. By extracting key features and using them as model inputs, traditional machine learning algorithms, such as linear regression, Support Vector Machine (SVM), etc., are used for predicting surface roughness. Çaydaş et al. [6] designed three distinct SVM algorithms for surface roughness prediction in stainless steel turning. They compared their performance with that of artificial neural networks (ANNs). The experimental results demonstrated that all three SVM algorithms significantly outperformed ANN in terms of prediction accuracy. Pimenov et al. [7] employed various machine learning algorithms to assess the influence of maximum wear, machining time, and cutting power on surface roughness prediction. The results indicated that the Random Forest and Regression Tree models achieved higher prediction accuracy for surface roughness. Siyambas et al. [8] utilized the XGBoost algorithm to predict the surface roughness of titanium alloys, aiming to optimize coolant selection for achieving the required product quality at minimal cost. Zhang et al. [9], considering industrial application demands such as applicability, implementation simplicity, and cost-effectiveness, developed a Gaussian Process Regression model to predict surface roughness in the turning process of brass.

Deep learning-based surface roughness prediction methods leverage end-to-end neural networks to automatically extract complex high-dimensional features through multi-layer structures. Such methods have also been widely used in industrial fields, including aerospace, automotive manufacturing, and precision parts machining [10,11]. For example, Pan et al. [12] utilized vibration signals as input for a Convolutional Neural Network (CNN) to develop a surface roughness prediction model. Guo et al. [13] implemented a Long Short-Term Memory (LSTM) network as the prediction framework, incorporating grinding force, vibration, and acoustic emission signals as inputs, achieving superior predictive performance. Lin et al. [14] proposed a surface roughness prediction framework that integrates three models: Fast Fourier Transform Deep Neural Network (FFT-DNN), Fast Fourier Transform Long Short-Term Memory Network (FFT-LSTM), and one-dimensional Convolutional Neural Network (1-D CNN). In this framework, FFT-DNN and 1-D CNN are used for feature extraction, and FFT-LSTM serves as the prediction model, yielding highly accurate surface roughness predictions.

In recent years, swarm intelligence algorithms have been increasingly combined with machine learning and deep learning to develop smarter and more efficient manufacturing solutions, aiming to optimize processes, improve prediction accuracy, and enhance decision-making. For instance, researchers have employed Grey Wolf Optimization (GWO) to fine-tune support vector machine (SVM) parameters [15], combined quantum-behaved particle swarm optimization (QPSO) with machine learning for surface defect prediction [16], and utilized genetic algorithm (GA)-optimized artificial neural networks (ANNs) to analyze machining effects on material surfaces [17]. Additionally, gradient-boosting regression trees (GBRTs) coupled with GA have been used to model process parameters and optimize predictive performance [18].

Both traditional machine learning and deep learning heavily rely on the quantity and quality of samples. However, real-world production often presents challenges such as high data collection costs, long cycle times, and complex and variable working conditions, making it difficult to obtain large-scale, high-quality data. Optimizing the representation of the feature space based on limited data and constructing high-accuracy prediction models with greater generalization capability are key issues in few-shot learning research. Virtual Sample Generation (VSG) is a primary solution in this field, which can generate new samples and expand the training dataset. Studies have shown that incorporating synthetic data can significantly improve the prediction accuracy and generalization ability of models [19]. This approach effectively addresses the issue of insufficient samples in environments with data imbalance or small-sized datasets, with particularly notable effects in tasks involving fewer samples [20]. In the field of surface roughness prediction, most existing studies focus on generating synthetic samples using deep learning methods or linear interpolation techniques. Wen et al. [21] proposed a local linear estimator for point-wise prediction in end milling, incorporating a pseudo-data generator to improve the fully interpolated local linear estimation performance. However, linear interpolation-based virtual sample generation methods can only create new samples among existing data points, restricting them to the range of known data distributions. Several studies have employed the adversarial training mechanism between generators and discriminators in Generative Adversarial Networks (GANs) to generate realistic synthetic data for training dataset augmentation, thereby enhancing model generalization capability. Wang et al. [22] introduced a data augmentation approach for surface roughness prediction based on a GAN, which enhanced vibration signal data and significantly improved prediction accuracy. Cooper et al. [23] designed a Conditional GAN to generate power signals associated with different process parameter combinations, effectively enhancing CNN-based surface roughness prediction accuracy.

In addition, most of the current research focuses on simple feature generation or the combination of existing features, which fails to adequately capture the deeper interactions between different variables. The exploration of systematic feature construction techniques remains insufficient.

Wang et al. employed a quadratic polynomial approach to expand machining parameter features [24]. While this method offers certain advantages, such as simplicity in form and computational efficiency, its fundamental limitation lies in its insufficient capability to represent nonlinear process features. Ruan et al. extended the machining parameter features using higher-order polynomials and introduced a variational autoencoder (VAE) to generate virtual samples to alleviate the issue of data scarcity [25]. Although higher-order polynomials enhance the expressive power compared to quadratic ones, they are still constrained by fixed functional forms, lack adaptability to feature structures, and suffer from an exponential increase in computational complexity with the order of the polynomial. Moreover, VAEs require large datasets to learn the true data distribution effectively, and under small-sample conditions, they tend to generate low-diversity or unrealistic samples, such as repeated or overly similar data. Chen et al. employed three different types of generative adversarial networks (GANs) to augment 1030 original machining data samples [26], but they still failed to adequately capture the true data distribution. Similarly, Brock et al. pointed out that the performance of GANs significantly degrades when trained on limited data [27]. Yu et al. attempted to mitigate overfitting under data scarcity by regularizing the discriminator in the GAN framework, but the generator remained unconstrained [28], which could result in the generation of samples that do not conform to process principles. In contrast, virtual sample generation shows greater potential under small-sample scenarios.

Therefore, using more advanced VSG-based feature construction strategies is essential in small-sample scenarios. These strategies help uncover additional useful information to improve the accuracy of surface roughness prediction. To address these existing challenges, further exploration of efficient and flexible methods for data generation and feature mining is necessary. On one hand, more tailored virtual sample generation strategies should be designed for different working conditions and data distribution characteristics to maximize the enrichment of the sample space. On the other hand, a more systematic feature expansion approach should be devised, focusing on deep variable correlations and nonlinear features, to fully explore the intrinsic mechanisms of the processing process and potential information. This will provide stronger support for predicting roughness in small-sample conditions.

To address the challenges mentioned above, this paper presents a novel data augmentation method, i.e., a Genetic Algorithm-based Virtual Sample Generation (GA-VSG) and a Genetic Programming-based Feature Construction (GP-FC) approach, for predicting surface roughness, which applies swarm intelligence algorithms to optimize the feature space for data enhancement.

By combining acceptance–rejection sampling with the crossover and mutation operations of the genetic algorithm, the method ensures that the generated samples maintain a distribution consistent with the original data at the feature level. Then, incorporating the principle of minimizing absolute information gain, the approach iteratively selects the optimal offspring samples, guaranteeing the authenticity of generated samples at the sample level. Subsequently, the GP approach is employed to conduct deep feature extraction from both original and virtual samples. By adaptively combining and transforming existing features, the method constructs more representative composite variables and preserves key information from the machining process. It also compensates for nonlinear residuals that are hard to capture in conventional feature spaces, thereby enhancing the accuracy and robustness of surface roughness prediction. Finally, an Extreme Random Trees Regressor (ET) model is used for the final prediction.

The key contributions of this paper are summarized as follows:

(1) We validate the authenticity of virtual samples in surface roughness prediction at both the sample and feature levels. Extensive experiments demonstrate the contribution of virtual samples in enhancing the generalization ability of the predictive model;
(2) Based on a residual compensation model, we adaptively construct features to extract deeper information from the data. This process generates more representative composite variables, effectively capturing nonlinear residuals and addressing the limitations of traditional machining parameter-based feature spaces;
(3) Using the SHAP (Shapley Additive Explanations) framework, we provide consistent and intuitive explanations for model predictions. We also identified the average total contribution of key features affecting surface roughness in AWJP and MJP.

The remainder of this paper is organized as follows. The Methods section introduces the proposed approach in detail. The Experimental Setup section describes the datasets and algorithm parameters. The Results and Discussion section presents a comprehensive analysis of the experimental results, demonstrating the effectiveness and robustness of the proposed method. Finally, the Conclusion section summarizes the key contributions of this work and discusses future research directions in interpretability and feature construction, offering insights for further improving the intelligence and accuracy of surface roughness prediction.

## 2. Materials and Methods

### 2.1. Method

**Figure 1:**
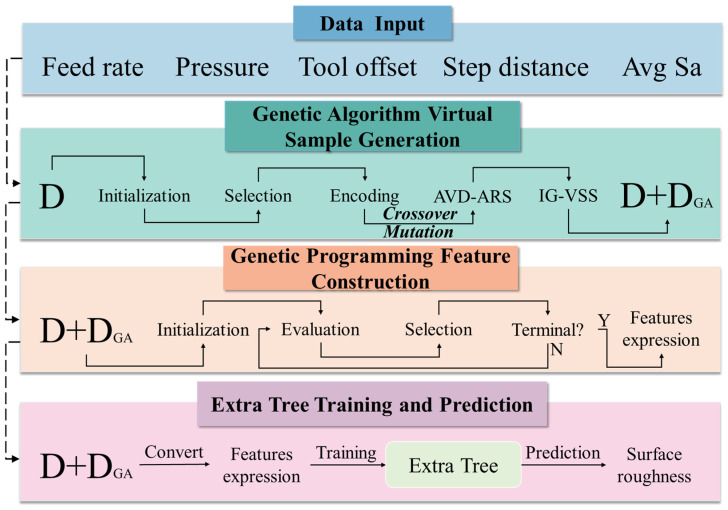
The framework of the VSG-FC model.

In this paper, a Genetic Algorithm-driven Virtual Sample Generation (GA-VSG) and a Genetic Programming-driven Feature Construction (GP-FC) method is proposed for surface roughness prediction, named as VSG-FC. Figure 1 provides an overview of this method, consisting of four main steps: data input, GA-based virtual sample generation, GP-based feature construction, and Extreme Random Tree regression (ET) training and prediction, which are explained in detail in the following:

(1) Data Input: Arithmetic mean surface roughness (Sa) is utilized as a feature alongside four additional features (Feed rate, Pressure, Tool set, Step distance) as input to the GA-VSG. This approach ensures that the generated virtual samples align with the real data in terms of the joint distribution of Sa and the other features, thereby enhancing the validity of the generated samples;
(2) The GA-VSG Module: The original dataset D serves as the input to GA. The process begins by initializing a GA population where each individual represents a potential virtual sample. Two parent samples are randomly selected and encoded using IEEE-754 floating-point format. The encoded samples undergo crossover and mutation operations. Following these operations, the generated new samples are evaluated through two key mechanisms: (1) Attribute-based Virtual Sample Discrimination using Acceptance–Rejection Sampling (AVD-ARS) to verify feature distribution consistency, and (2) Information Gain-based Virtual Sample Selection (IG-VSS) to ensure sample distribution authenticity. This evolutionary cycle repeats iteratively until the desired quantity of virtual samples is generated, through which the dataset $D_{GA}$ is obtained;
(3) The GP-FC Module: The datasets D and $D_{GA}$ are utilized as inputs to the GP. The GP operation begins by initializing a population where each individual represents a mathematical expression for feature transformation. Each GP individual is evaluated using a residual compensation model, with the Lexicase selection operator applied to identify high-performing individuals. The selected individuals then undergo crossover and mutation to generate new offspring. This evolutionary process iterates until meeting the maximum iteration criterion, at which point the best-performing GP individual is selected as the final feature construction strategy;
(4) The ET training and prediction: Finally, the GP-generated optimal feature construction strategy is used to map the original and dummy samples into a new feature space, which serves as the input to the ET module and outputs the final surface roughness prediction.

#### 2.1.1. GA-VSG Framework

**Figure 2:**
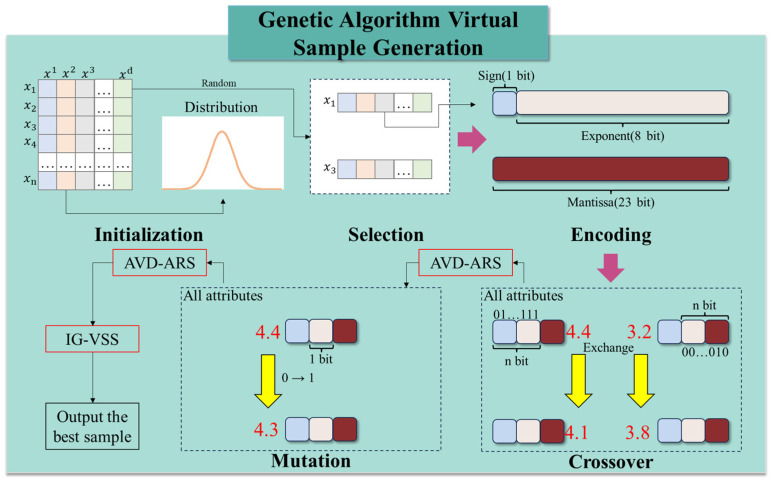
The GA-VSG framework.

Yu et al. [29] proposed a GA-VSG algorithm, which ensures the authenticity of generated samples at both the sample level and feature level. Figure 2 illustrates the workflow of GA-VSG sample generation, which consists of the following steps: initialization, Parent Selection, IEEE-754 [30] Encoding, Crossover and Mutation, AVD-ARS, and IG-VSS.

Let $X=\{x_{1},x_{2}{,\ldots \ldots ,x}_{n}\}$ represent a dataset containing N samples, where each sample $x_{i}=\{x_{i}^{1},x_{i}^{2},\ldots \ldots ,x_{i}^{d}\}$ consists of d features. Determination of the number of virtual generated samples β and the mutation rate λ consists of two core operations: (1) initializing fundamental parameters and computing the entropy of the original dataset X to facilitate IG-VSS invocation, and (2) performing Gaussian distribution fitting for each column $x_{i}^{d}$ of the small original dataset to support the AVD-ARS operation. Then, two parent samples are randomly selected from the original dataset D to generate virtual samples in each iteration. Next, two parent samples $x_{1}$ and $x_{3}$ are randomly selected and encoded using the IEEE-754 floating-point format.

The encoded samples undergo crossover and mutation operations. This study employs single-point crossover, where the encoded feature values are randomly exchanged between two parent samples. Additionally, a single-point mutation is applied by flipping a randomly selected bit (0 to 1 or 1 to 0) in a parent sample.

After these operations, the AVD-ARS operation is applied to evaluate whether the newly generated feature values conform to the Gaussian distribution of the original samples. A newly generated sample proceeds to the next step only if all its feature values meet the distribution criteria; otherwise, it undergoes another round of crossover or mutation. In the AVD-ARS, ARS is an efficient Monte Carlo sampling method designed to generate a sample set x that matches a given probability density function p(x) within a known distribution δ. To achieve this, a proposal distribution G with a known probability density function g(x) is selected. A constant C is set to satisfy Equation (1). ARS then samples x from G and selects a random number μ from a uniform distribution in [0,1]. If Equation (2) holds, x is accepted as a valid sample from δ.

(1) $$C\times g\left(x\right)\geq p\left(x\right)$$

(2) $$\frac{p(x)}{C\times g(x)}\geq \mu$$

Each pair of parent samples produces two offspring, from which the IG-VSS operation selects the optimal offspring sample. This process is repeated until the required number of virtual samples is generated.

In the IG-VSS, the information entropy of the original dataset D is assumed to be H. When a new sample is added to D, the entropy is updated to H+△h. A high △h indicates that the new sample has a low probability under the original distribution of D, meaning it is not well-matched to the dataset and should be discarded. Instead, virtual samples with lower △h should be selected. The K-nearest neighbors (K-NN) nonparametric entropy estimation method is employed to compute information entropy, as given in Equation (3). The change in entropy △h is computed by Equation (4), where s represents the newly added sample. Finally, the virtual sample with the smallest △h is selected as a valid virtual sample.

(3) $$H\left(D\right)\approx -\frac{1}{N}\sum_{i=1}^{N}{log}^{p(x_{i})}$$

(4) $$△h=|H\left(D\right)-H\left(D\cup s\right)|$$

#### 2.1.2. GP-FC Model

**Figure 3:**
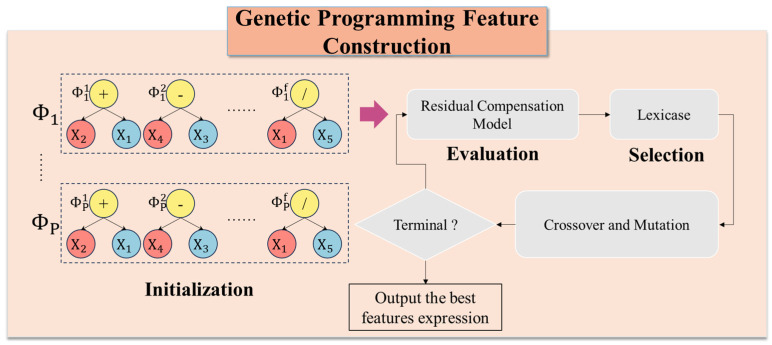
The flowchart of the GP-FC operation.

Figure 3 presents the flowchart of the GP-FC operation, which includes initialization, fitness value evaluation, selection, crossover, and mutation. In the GP-FC operation, an individual is a set of f trees, with each tree Φ corresponding to a mathematical expression. As a whole, an individual represents a set of feature construction strategies. $\Phi_{1}$ is composed of the mathematical expressions $\Phi_{1}=\{\Phi_{1}^{1},\Phi_{1}^{2},\ldots \Phi_{1}^{f}\}$. The process begins with initialization, during which a population of P individuals is generated, forming a complete initial population, and then the crossover rate υ and mutation rate ξ are determined.

**Figure 4:**
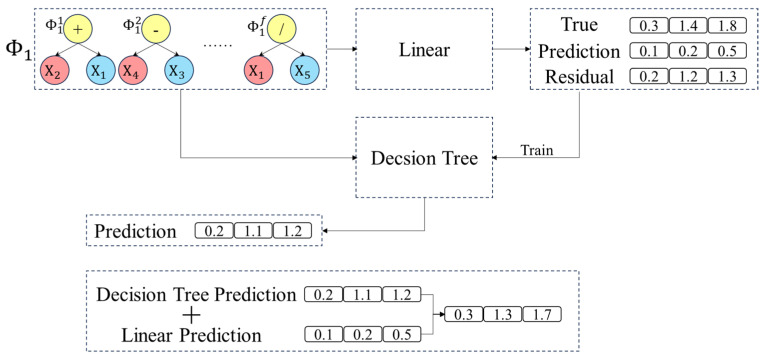
Flowchart of the residual compensation model.

In the GP-FC, an individual represents a set of feature construction strategies that map the original feature space to a new feature space defined by a mathematical expression. Therefore, after generating an individual, the effectiveness of these feature construction strategies must be evaluated using a model. In traditional GP algorithms, individual evaluation typically relies on a single linear or nonlinear model. However, complex interactions can arise during the feature construction process. To address this issue, a residual compensation-based evaluation model is proposed to assess the performance of each GP individual. Specifically, a linear regression model is first trained to fit the linear component of the data, followed by a decision tree regression model to fit the residuals of the linear regression model, i.e., the nonlinear component. The final prediction is obtained by combining the outputs of both models. As illustrated in Figure 4, the data first undergo feature reconstruction using the feature selection strategy provided by an individual $\Phi_{1}$ to map the original feature space to a new feature space. A linear regression model is then trained using the feature-reconstructed data. Next, the training samples are predicted using the linear regression model, and the residuals are computed. Subsequently, a decision tree regression model is trained using both the feature selection strategy corresponding to $\Phi_{1}$ and the obtained residuals. Finally, the results obtained from both the linear regression model and the decision tree regression model are combined to produce the final predictions of the test samples.

The Mean Squared Error (MSE) is employed to evaluate the performance of each mathematical expression, which also serves as the individual’s fitness value. It is worked out by Equation (5), where n is the number of samples, $y_{i}$ is the actual value, and ${\hat{y}}_{i}$ is the corresponding predicted value. A lower MSE indicates better predictive performance of the expression.

(5) $$MSE=\frac{1}{n}\sum_{i=1}^{n}{\left(y_{i}-{\hat{y}}_{i}\right)}^{2}$$

The Lexicase selection method is adopted to enhance feature diversity and adaptability. Predictions are made on the GP validation set, and the Square Error (SE) is computed for each sample. The prediction errors for all validation samples are concatenated into a vector of shape n×1, where n is the number of samples in the GP validation set. Each individual corresponds to an error vector $E_{L}=\{e_{1},e_{2},\ldots \ldots ,e_{n}\}$. Then a random index κ is selected from [1, *n*], and the Median Absolute Deviation (MAD) is computed as ${mad}_{\kappa }=median(|e_{n}-median(E_{L})|)$. Individuals with $e_{\kappa }$ values smaller than ${mad}_{\kappa }$ are retained, while the rest are discarded. This process is repeated until only one individual remains, which is then added to the parent pool.

**Figure A1:**
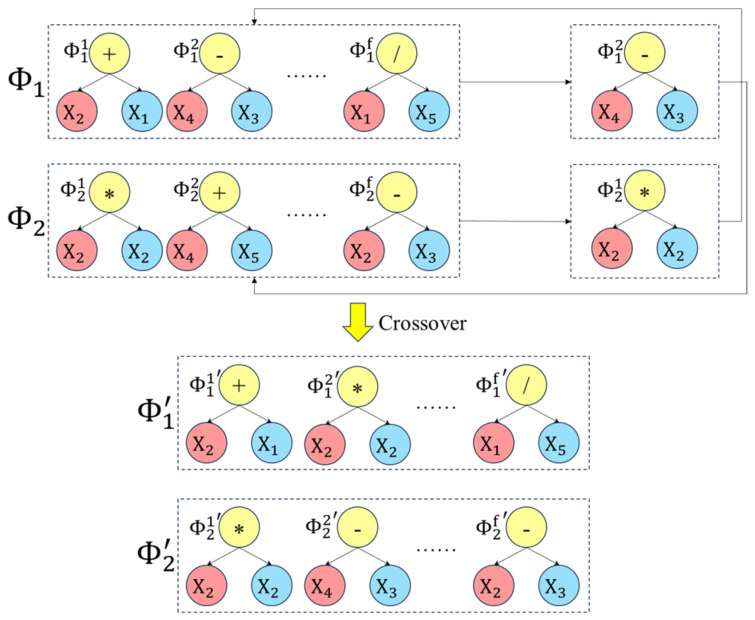
The operation of crossover, where $\Phi_{1}$ and $\Phi_{2}$ represent the two parents and ${\Phi_{1}}^{\prime }$ and ${\Phi_{2}}^{\prime }$ represent the two offsprings.

**Figure A2:**
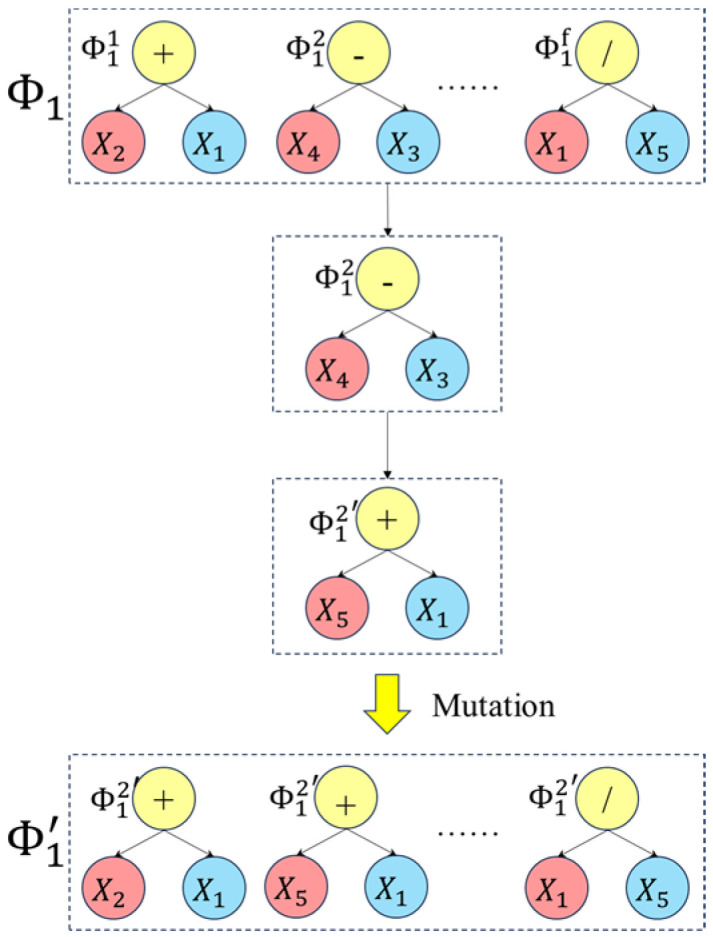
The operation of mutation, where $\Phi_{1}$ represents the parent and ${\Phi_{1}}^{\prime }$ represents the offspring.

After the selection process, each parent undergoes crossover or mutation based on a given probability. Finally, when the maximum number of GP iterations is reached, the individual with the lowest MSE is selected as the optimal individual. The detailed process is illustrated in Figure A1 and Figure A2.

#### 2.1.3. Extreme Random Tree Training and Prediction

Finally, the effectiveness of data enhancement is verified using the Extreme Random Tree algorithm [31], as detailed in Algorithm A1. The Extreme Random Tree algorithm is a variant of the Random Forest, where the final regression output is obtained by constructing multiple decision trees and aggregating the predictions from all the trees. This method enhances the model’s generalization ability and computational efficiency.

### 2.2. Materials

#### 2.2.1. Experimental Dataset

The effectiveness of the proposed algorithm was validated on datasets obtained from two different polishing methods. Traditional process parameter optimization in polishing typically relies on extensive trial-and-error experiments, which are time-consuming, labor-intensive, and difficult to scale for mass production. Therefore, developing high-accuracy surface roughness prediction models is of significant practical importance for enabling intelligent process optimization and reducing production costs. The experiments were conducted on a ZEEKO IRP200 polishing machine (ZEEKO Ltd., Coalville, UK).

The first experimental dataset was obtained from the Abrasive Water Jet Polishing (AWJP) of 3D-printed CoCr alloy, with 3D-printed CoCr alloy specimens serving as the test objects. Each specimen has a dimension of 42 mm × 40.5 mm × 10 mm, and a standardized polishing area of 6 mm × 3 mm was designated for each experiment. A 1000# alumina slurry (10 wt%) manufactured by Fujimi Corporation, Kiyosu, Japan, was used as the polishing medium, and the slurry was delivered through a 1 mm diameter sapphire nozzle impacting the surface vertically. We focused on investigating the effects of key process parameters, while other parameters were kept constant. After polishing, surface roughness was measured using a ZYGO Nexview white light interferometer.

The surface roughness was evaluated using a ZYGO Nexview optical profiler (manufactured in the Middlefield, CT, USA) white light interferometer with a 40× objective lens. The instrument provided lateral and vertical resolutions of 208.8 nm and 0.1 nm, respectively. For each specimen surface, three randomly selected locations were measured, with each measurement covering an area of 213.78 μm × 213.78 μm. Surface quality was quantified using the arithmetic mean roughness (Sa) in accordance with ISO 25178 standards [32]. Data processing was performed using the Mx software package, where a ninth-order polynomial filter was applied in the analysis. All other parameters maintained the software’s default configuration [33]. Detailed experimental parameters are presented in Table 1. Under varying polishing conditions, a total of 40 data samples were collected, as shown in Table 2.

The second dataset was obtained by applying Multi-Jet Polishing (MJP) to 3D-printed 316L stainless steel components. Using specimens designed with a dimension of 10 mm × 10 mm × 10 mm. A seven-nozzle polishing unit was employed. Through a systematic experimental design, polishing data were collected under 43 different combinations of process parameters. The main variables included feed rate, fluid pressure, tool offset, and step-distance. The experimental parameters are presented in Table 3, and the final experimental results are summarized in Table 4.

#### 2.2.2. Parameters of the Proposed Method

In GA-VSG, two key parameters are defined: the number of virtual samples β and the mutation rate λ. The value of β is set equal to the number of training samples to expand the dataset while preserving the original data distribution and statistical properties as much as possible. The mutation rate λ is set to 0.01.

The parameter settings for GP are detailed in Table 5. The number of Trees determines the quantity of features generated, while Functions represent the operators between features within a GP individual.

MSE is used as the default evaluation metric to compare different algorithms and parameter settings. A total of 80% of the data are allocated as the training set and 20% as the test. Each algorithm is evaluated on each dataset using five different random seeds.

#### 2.2.3. The Division of Training Data and Test Data

The dataset is randomly partitioned into training and test sets at a 4:1 ratio, with the final performance of all algorithms evaluated solely based on the test set. During the fitness evaluation phase of the GP-FC algorithm, the training set was further divided into model training data and model validation data at a 3:1 ratio. The model training data was used to train the residual model, while the model validation data served to evaluate the fitness values of GP individuals.

#### 2.2.4. Benchmark Algorithms

The proposed method is compared with eight state-of-the-art machine learning techniques in this study:

SVR [34]: Support Vector Regression, which fits the data by maximizing the margin;

RF [35]: Ensemble learning methods, which improve predictive performance by constructing multiple decision trees and aggregating results through voting or averaging;

GBM [36]: It constructs a series of weak learners by iteratively optimizing residuals;

XGB [37]: An efficient and scalable implementation of gradient boosting trees that supports parallel computation and regularization;

MLP [38]: A feedforward neural network that learns complex patterns through multiple layers of nonlinear transformations;

DT [39]: A tree-based classification or regression method that generates decision rules by recursively partitioning the data;

KNN [40]: A non-parametric method that makes predictions based on the voting or averaging of nearby samples using a distance metric;

CAT [41]: A gradient boosting algorithm designed for efficient handling of categorical features, with built-in support for automatic categorical variable processing.

Additionally, the proposed approach is compared with two state-of-the-art sample generation methods, which are as follows:

GAN [42]: An adversarial framework consisting of a generator and a discriminator. The generator aims to produce realistic data, while the discriminator attempts to distinguish real data from the generated data. Both components are optimized through adversarial training;

VAE [43]: A generative model that maps data into a latent space using an encoder and reconstructs it through a decoder. It incorporates variational inference to optimize the distribution of latent variables.

## 3. Results and Discussion

### 3.1. Validation of the GA-VSG Method

The MSE of different machine learning models on the two original experimental datasets was first compared. The results are presented in the Base column of Table 6. On AWJP, Random Forest (RF) delivers the best performance with an MSE of 0.1509, significantly outperforming the other models. ET and XGB follow closely behind with MSEs of 0.1522 and 0.1674, respectively, underscoring the effectiveness of ensemble learning methods on this dataset. On MJP, GBM achieves the best performance, with an MSE of 0.0011, demonstrating its strong capability in capturing fine-grained data patterns. XGB and RF also perform well, both with an MSE of 0.0014, further confirming the superiority of ensemble learning methods on complex datasets. The experimental results from both datasets indicate that ensemble learning methods consistently outperform single models in terms of MSE. This suggests that ensemble methods effectively capture complex nonlinear relationships and interaction features by aggregating predictions from multiple weak learners. Additionally, the performance of the same machine learning method varies slightly across different datasets. This variation may stem from intrinsic dataset characteristics and differences in data processing methods.

Then, the MSE of different machine learning models on the two experimental datasets was compared, which were augmented with data generated by the GA-VSG method. The arrow ↑ indicates an increase in MSE, ↓ indicates a decrease in MSE. After applying GA-VSG, the MSE of most machine learning methods on AWJP significantly decreases, with an average decrease of 0.0374, indicating that GA-VSG effectively expands the training set by generating virtual samples and enhancing model generalization. Notably, KNN’s MSE decreases by 11.59% and ET by 3.35%. DT, RF, and XGB experience a slight increase in MSE, likely because the virtual samples generated by GA-VSG alter the original data distribution. On MJP, GA-VSG increases the MSE of CAT and DT, while the MSE of other machine learning methods either decreases or remains unchanged, with an average decrease of 0.0068. This further demonstrates that the effectiveness of virtual sample generation highly depends on both the characteristics of the machine learning method and the dataset. For instance, CAT and DT may be more sensitive to noise or distribution shifts introduced by virtual samples, whereas other methods can better leverage the expanded dataset, improving or maintaining their performance.

Overall, GA-VSG proves effective for most machine learning methods, as the generated virtual samples successfully enhance model generalization. GA-VSG generates virtual samples through crossover and mutation operations of genetic algorithms, which essentially perform reasonable interpolation within high-density regions of the original data distribution. This difference explains why KNN (which relies on local similarity) and ET (which mitigates overfitting through additional randomness) exhibit improved performance, while DT-based models show increased sensitivity due to their rigid splitting rules. The accuracy improvement observed with GA-VSG in both AWJP and MJP suggests that the data in these domains possess smooth and continuous characteristics, making them suitable for interpolation-based augmentation.

### 3.2. Comparison with Other Virtual Sample Generation Methods

As shown in Table 7, bold indicates the method with the greatest reduction in MSE. The GA-VSG method is compared with two other state-of-the-art virtual sample generation methods: GAN and VAE. Across both datasets, GA-VSG significantly reduces the MSE of most machine learning methods, indicating that its generated virtual samples effectively expand the training set and enhance model generalization. Overall, GA-VSG outperforms both GAN and VAE on both datasets, particularly in improving model generalization and reducing MSE. Therefore, GA-VSG proves to be a more effective virtual sample generation method, making it well-suited for various machine learning tasks.

### 3.3. Validation of VSG-FC Effectiveness

**Figure 5:**
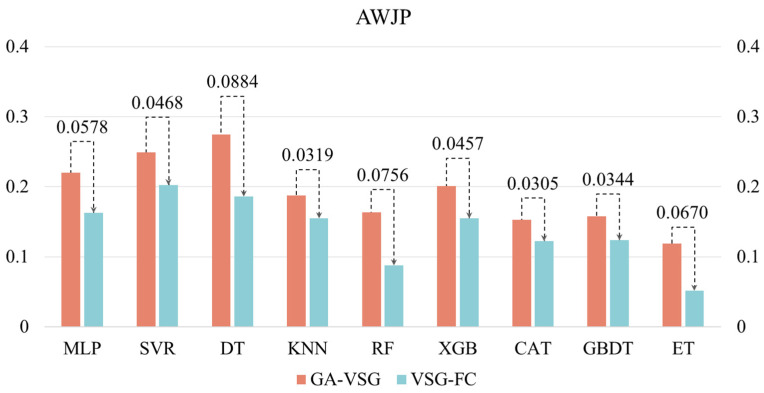
Validation of VSG-FC effectiveness AWJP dataset.

**Figure 6:**
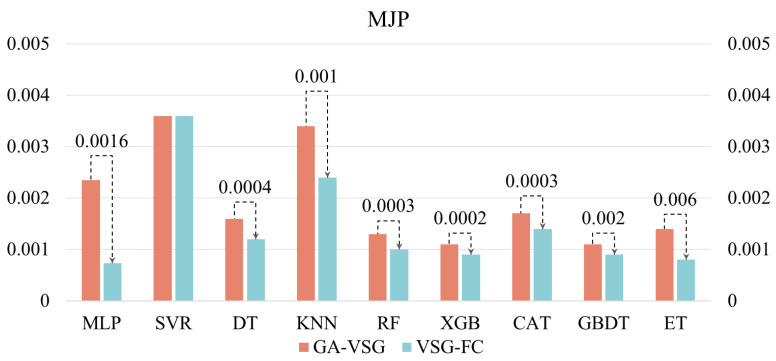
Validation of VSG-FC effectiveness MJP dataset.

After generating virtual samples using the GA-VSG method, the feature space representation of the data is further enhanced through the GP-FC feature construction approach. To evaluate the effectiveness of GP-based feature construction, the performance of different machine learning methods before and after applying the GP-FC method was compared. The experimental results shown in Figure 5 and Figure 6 indicate that VSG-FC consistently outperforms GA-VSG across all datasets and models. On AWJP, VSG-FC significantly reduces MSE for all models compared to GA-VSG (to facilitate a clearer and more intuitive performance comparison of VSG-FC, all MLP values in Figure 6 have been uniformly scaled by a factor of 10^2^). For example, in MLP, MSE decreases from 0.2204 to 0.1626, a reduction of 26.2%. In ET, MSE drops from 0.1188 to 0.0518, reducing by 56.4%. These results demonstrate that VSG-FC provides substantial performance improvements, especially for complex datasets. On MJP, VSG-FC also delivers strong results. While the MSE of SVR remains unchanged at 0.0036, all other models show reductions in MSE. For example, in DT, MSE decreases from 0.0016 to 0.0012, a reduction of 25%, and in ET, MSE drops from 0.0014 to 0.0008, a reduction of 42.9%. These results suggest that VSG-FC is highly adaptable across different types of datasets.

While GA-VSG improves model generalization through virtual sample generation, its performance gains are limited. In contrast, VSG-FC further optimizes the model through feature construction, leading to a more substantial performance improvement. Different models respond to VSG-FC with varying degrees of improvement. For instance, on AWJP, ET achieves the largest MSE reduction of 56.40%, whereas on MJP, MLP shows the most significant improvement (MSE reduction of 68.63%). This suggests that the optimization effect of VSG-FC varies across models, likely due to differences in model structure and learning capacity. Its core idea is to capture underlying patterns in the data by constructing new features, thereby compensating for the limitations of the original features. By comparing the MSE of GA-VSG and VSG-FC across different datasets and models, the experiment results validate the effectiveness of VSG-FC in reducing MSE across different datasets and models.

### 3.4. Shapley-Based Interpretability Analysis

Shapley theory [44] is used for fairly distributing contributions among participants in cooperative games. In machine learning, Shapley theory is widely applied in model interpretability analysis, particularly through the SHAP (Shapley Additive Explanations) framework, which calculates SHAP values to provide consistent and intuitive explanations for model predictions [45]. In this study, the Shapley method is employed to visualize the contributions of different features in influencing the prediction of surface roughness, providing a theoretical reference for subsequent experiments.

**Figure 7:**
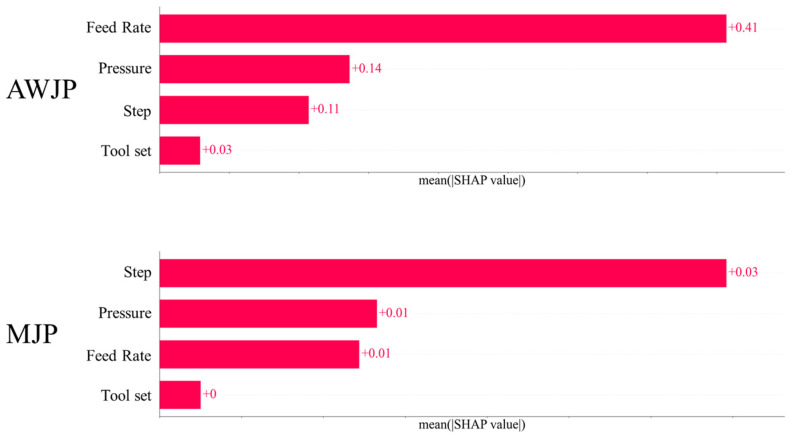
Bar chart of the Shapley contribution for different features.

Figure 7 presents the average total contribution of four key features affecting the surface roughness in AWJP and MJP. The second and fourth most influential features in both datasets are Pressure and Tool set, respectively. This difference may be attributed to variations in process conditions, machining environments, or differences in the distribution of input features across datasets.

In AWJP, the SHAP value of Feed Rate is significantly higher than that of other features, indicating its dominant influence on surface roughness prediction. Feed Rate directly affects the material removal rate and the contact time between the tool and the workpiece during machining. A higher Feed Rate may lead to increased cutting forces and intensified vibrations, resulting in a rougher surface. Conversely, a lower Feed Rate may cause extended friction time, leading to heat accumulation, which can also deteriorate surface quality. Processing pressure may influence the contact between the tool and the workpiece. Insufficient pressure can result in incomplete cutting, reducing machining efficiency, while excessive pressure can accelerate tool wear, indirectly degrading surface quality.

In MJP, the SHAP value of Step dominates, indicating that Step is the key control parameter for surface roughness. Step-over determines the overlap ratio of adjacent machining paths. A larger step-over may leave unprocessed areas (e.g., tool marks in milling), directly increasing surface roughness, while a smaller step-over may lead to material hardening or tool wear due to repeated cutting.

The average surface roughness in MJP is lower than that in AWJP, which may be a key factor contributing to the difference in feature importance. This suggests that for workpieces with higher surface roughness, Feed Rate should be the primary focus, whereas for those with lower roughness, Step is more critical.

**Figure 8:**
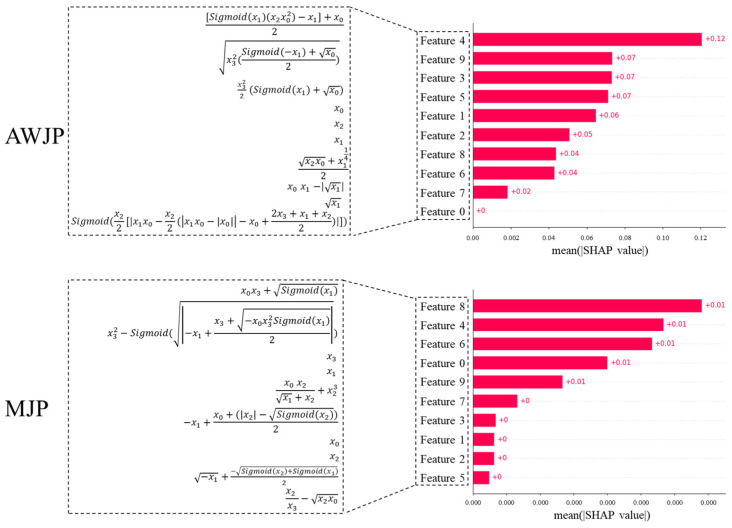
Bar chart of the Shapley contribution for features of VSG-FC.

Additionally, Figure 8 illustrates the contributions of the VSG-FC features in AWJP and MJP, where $x_{0}$, $x_{1}$, $x_{2}$, and $x_{3}$ represent Feed Rate, Pressure, Tool Set, and Step, respectively. VSG-FC constructs different feature expressions across the two datasets to represent surface roughness prediction. Compared to the original machining parameters, the features constructed by VSG-FC exhibit superior predictive performance. In the AWJP dataset, $x_{0}$: Feed Rate remains the dominant feature in forming higher-order features, which aligns with the SHAP values calculated from the original features. Similarly, in the MJP dataset, $x_{3}$, Step is the leading feature.

VSG-FC not only significantly enhances prediction performance through feature construction but also generates structured and interpretable expressions that hold greater value for industrial research. Unlike traditional black-box feature construction methods, this approach outputs explicit mathematical forms that can be directly linked to physical machining mechanisms, thereby providing a traceable analytical path for process optimization. These symbolic features offer triple potentials: (1) a tool for discovering process knowledge, enabling the extraction of latent physical laws from data; (2) to build a general feature library across different processes, reducing the cost of repeated modeling; (3) to support causal inference studies, by enabling reverse analysis of key process parameter relationships through the expressions.

### 3.5. Discussion

We have systematically evaluated the performance of various machine learning models on AWJP and MJP datasets, with an in-depth investigation into how virtual sample generation and feature engineering methods affect model generalization capabilities. Experimental results demonstrated that ensemble learning methods exhibit significant advantages in both tasks, validating their effectiveness in capturing complex data features through multi-learner collaborative mechanisms. Notably, the models showed clear task-dependent performance characteristics. For instance, RF achieved optimal performance on AWJP data while GBM outperformed on MJP data.

Regarding data augmentation, the proposed GA-VSG method employs a genetic algorithm-driven intelligent interpolation strategy to effectively expand training samples while preserving original data distribution characteristics. Compared with generative approaches like GAN and VAE, GA-VSG demonstrates more stable performance improvements, particularly showing significant gains with KNN and ET models, thereby offering new insights for addressing small-sample learning challenges. However, the sensitivity of decision tree-based models to virtual samples also reveals limitations of data augmentation techniques, indicating their effectiveness is constrained by both model structural robustness and data distribution smoothness. The further developed VSG-FC feature construction method, which optimizes feature space through GP, outperformed GA-VSG across all tested models, confirming the synergistic effects between feature engineering and data augmentation.

By analyzing the Shapley values of features computed based on model predictions, the varying importance of process parameters within the model was revealed, providing a theoretical foundation for understanding quality control mechanisms under different machining conditions. The dominant influence of feed rate in AWJP versus the crucial role of step parameters in MJP reflects differential impact patterns of distinct machining mechanisms on surface morphology. These findings not only validate expectations from traditional machining theory but also provide a quantitative basis for feature selection in intelligent process optimization systems.

## 4. Conclusions

In the field of machining, surface roughness prediction plays a crucial role in optimizing manufacturing processes, improving product quality, reducing production costs, and ensuring that components meet specific functional and assembly requirements. In this study, we propose the VSG-FC framework, a surface roughness prediction model that integrates GA-VSG and GP-FC. This framework enhances the data space from two perspectives—sample augmentation and feature construction—to improve model prediction accuracy and generalization capability. The GA-VSG method is first used to generate high-quality virtual samples, significantly expanding the training dataset and addressing the issue of data scarcity in industrial polishing processes. Then, the GP-FC method enhances the model’s learning capacity by automatically constructing discriminative feature combinations. Specifically, the GA-VSG approach overcomes the limitation of deep learning models that rely heavily on large-scale real-world datasets. By leveraging evolutionary search, it effectively enriches the data distribution and reduces dependence on physical data collection. In addition, GP-FC automatically searches for optimal feature combinations, reducing the reliance on domain-specific knowledge for manual feature engineering and enabling end-to-end feature optimization.

The effectiveness of the VSG-FC framework is validated on two real-world polishing process datasets. Experimental results demonstrate that GA-VSG substantially improves the performance of most machine learning models, reducing MSE by an average of 0.0374 on the AWJP dataset and by 0.0068 on the MJP dataset, confirming the effectiveness of evolutionary-based data augmentation. Furthermore, GA-VSG outperforms generative models such as GAN and VAE in enhancing predictive performance and generalization, as its virtual samples lead to more accurate and robust model outputs. When GA-VSG is combined with GP-FC, the resulting VSG-FC model shows a significant performance boost—on the AWJP dataset, the ET model achieved a maximum MSE reduction of 56.40%, while on the MJP dataset, the MLP exhibited the most notable improvement with a 68.63% reduction in MSE. Additionally, Shapley value-based analysis identifies key features that drive surface roughness in different datasets and reveals their underlying mechanisms. This comparative analysis not only supports the theoretical influence of process parameters but also provides a basis for targeted optimization.

Future work will focus on two directions: algorithm optimization and application extension. On the algorithmic side, future efforts will introduce mechanisms such as expression compression, evolution strategies guided by regularization, or complexity penalty terms to encourage GP to construct feature expressions that are structurally simpler and semantically clearer, thereby enhancing model interpretability. On the application side, we plan to extend the proposed framework to other machining scenarios in order to comprehensively assess its generalization capability.

## Figures and Tables

**Table 1:** Parameter settings of the AWJP experiments.

Parameter	Range
Feed rate	10, 15, 20, 25, 30, 40, 60, 80 mm/min
Fluid pressure	4, 5, 6, 7, 8, 9, 10 bar
Tool offset	4, 6, 8, 10, 12, 14 mm
Step distance	4, 6, 8, 10, 12, 14 mm

**Table 2:** AWJP experiment data.

Number	Feed Rate (m/min)	Pressure (bar)	Tool Set (mm)	Step (mm)	Sa (μm)
1	10	8	8	0.3	0.3362
2	20	8	8	0.3	0.5633
3	30	8	8	0.3	0.8086
4	40	8	8	0.3	1.6408
5	60	8	8	0.3	2.3625
6	80	8	8	0.3	2.2951
7	10	5	8	0.3	0.4358
8	10	6	8	0.3	0.6905
9	10	7	8	0.3	0.1513
10	10	8	8	0.3	0.2125
11	10	9	8	0.3	0.2211
12	10	10	8	0.3	0.2091
13	10	8	4	0.3	0.2547
14	10	8	6	0.3	0.2231
15	10	8	8	0.3	0.1800
16	10	8	10	0.3	0.2999
17	10	8	12	0.3	0.2727
18	10	8	14	0.3	0.6423
19	10	8	8	0.1	0.1034
20	10	8	8	0.2	0.1443
21	10	8	8	0.3	0.3773
22	10	8	8	0.4	0.2967
23	10	8	8	0.5	0.8901
24	10	8	8	0.6	1.4509
25	4	10	6	0.1	0.3798
26	4	15	8	0.2	1.6405
27	4	20	10	0.3	2.1245
28	4	25	12	0.4	2.5081
29	6	10	8	0.3	0.6506
30	6	15	6	0.4	1.7621
31	6	20	12	0.1	0.3846
32	6	25	10	0.2	1.1872
33	8	10	10	0.4	0.3705
34	8	15	12	0.3	0.3694
35	8	20	6	0.2	0.5596
36	8	25	8	0.1	0.6685
37	10	10	12	0.2	0.1065
38	10	15	10	0.1	0.0774
39	10	20	8	0.4	0.4767
40	10	25	6	0.3	0.5114

**Table 3:** Parameter settings of the MJP experiments.

Parameter	Range
Feed rate	10, 15, 20, 25, 30, 40, 60, 80 mm/min
Fluid pressure	4, 5, 6, 7, 8, 9, 10 bar
Tool offset	2.5, 5, 7.5, 10, 12.5, 15 mm
Step distance	0.1, 0.2, 0.3, 0.4, 0.5, 0.6, 0.7, 0.8 mm

**Table 4:** MJP experiment data.

Number	Feed Rate (mm/min)	Pressure (bar)	Tool Set (mm)	Step (μm)	Sa (nm)
1	10	8	5	0.2	0.021
2	20	8	5	0.2	0.030
3	30	8	5	0.2	0.032
4	40	8	5	0.2	0.088
5	60	8	5	0.2	0.127
6	80	8	5	0.2	0.144
7	20	4	5	0.2	0.088
8	20	5	5	0.2	0.085
9	20	6	5	0.2	0.083
10	20	7	5	0.2	0.039
11	20	8	5	0.2	0.039
12	20	9	5	0.2	0.037
13	20	10	5	0.2	0.033
14	20	8	2.5	0.2	0.027
15	20	8	5	0.2	0.040
16	20	8	7.5	0.2	0.032
17	20	8	10	0.2	0.072
18	20	8	12.5	0.2	0.026
19	20	8	15	0.2	0.036
20	20	8	5	0.1	0.031
21	20	8	5	0.2	0.043
22	20	8	5	0.3	0.047
23	20	8	5	0.4	0.085
24	20	8	5	0.5	0.098
25	20	8	5	0.6	0.138
26	20	8	5	0.7	0.099
27	20	8	5	0.8	0.131
28	10	5	2.5	0.2	0.044
29	15	5	5	0.4	0.146
30	20	5	7.5	0.6	0.183
31	25	5	10	0.8	0.229
32	10	6	5	0.6	0.076
33	15	6	2.5	0.8	0.151
34	20	6	10	0.2	0.065
35	25	6	7.5	0.4	0.165
36	10	7	7.5	0.8	0.121
37	15	7	10	0.6	0.111
38	20	7	2.5	0.4	0.086
39	25	7	5	0.2	0.066
40	10	8	10	0.4	0.061
41	15	8	7.5	0.2	0.03
42	20	8	5	0.8	0.133
43	25	8	2.5	0.6	0.144

**Table 5:** Parameter settings for Genetic Programming.

Parameter	Value
Population size (P)	100
Maximal Generations	100
Crossover (υ)	0.9
Mutation (ξ)	0.1
Functions	+, -, *, /, sqrt, abs, negative, sigmoid
Number of Trees	10

**Table 6:** Validation of the GA-VSG effectiveness.

Method	AWJP			MJP		
	Base	GA-VSG	Difference	Base	GA-VSG	Difference
MLP	0.2447	0.2204	0.0244 ↓	0.2619	0.2349	0.0270 ↓
SVR	0.2760	0.2493	0.0268 ↓	0.0036	0.0036	0
DT	0.2435	0.2745	0.0311 ↑	0.0015	0.0016	0.0001 ↑
KNN	0.3032	0.1873	0.1159 ↓	0.0034	0.0034	0
RF	0.1509	0.1633	0.0124 ↑	0.0014	0.0013	0.0001 ↓
XGB	0.1674	0.2008	0.0334 ↑	0.0014	0.0011	0.0003 ↓
CAT	0.1684	0.1532	0.0153 ↓	0.0017	0.0017	0.0001 ↑
GBDT	0.1667	0.1581	0.0086 ↓	0.0011	0.0011	0
ET	0.1522	0.1188	0.0335 ↓	0.0015	0.0014	0.0001 ↓
Mean	0.2081	0.1917	0.0164 ↓	0.0308	0.0278	0.0003 ↓

**Table 7:** Comparison with other virtual sample generation methods.

Method	AWJP			MJP		
	GAN	VAE	GA-VSG	GAN	VAE	GA-VSG
MLP	0.2664	0.2710	**0.2204**	0.1839	**0.1488**	0.2349
SVR	0.2903	0.3246	**0.2493**	0.0193	0.0190	**0.0036**
DT	0.5903	**0.1246**	0.2745	0.0017	0.0237	**0.0016**
KNN	0.3041	0.2589	**0.1873**	0.0115	0.0164	**0.0034**
RF	0.2350	**0.1033**	0.1633	0.0014	0.0134	**0.0013**
XGB	0.1807	**0.0775**	0.2008	0.0013	0.0163	**0.0011**
CAT	0.1997	0.2469	**0.1532**	**0.0012**	0.0150	0.0017
GBDT	0.1933	**0.0895**	0.1581	**0.0010**	0.0197	0.0011
ET	0.1580	0.1778	**0.1188**	0.0024	0.0129	**0.0014**

## Data Availability

Data are contained within the article.

## Appendix A

### Appendix A.3. ET Algorithm Process

During the construction of each decision tree, the Extreme Random Tree randomly selects a subset of features for node splitting and introduces additional randomness through Extra Trees. Unlike traditional decision trees, which search for the optimal splitting point, the Extreme Random Tree randomly selects several candidate splitting points and then chooses one randomly as the final splitting point. This added randomness reduces computational costs while increasing model diversity. Based on these randomly selected features and split points, the decision tree is built recursively until the stopping criteria are met, such as reaching the maximum depth or having fewer samples in the nodes than a predefined threshold.

**Algorithm A1.** ET
1. Input training dataset $D=\left\{\left(x_{1},y_{1}\right),\left(x_{2},y_{2}\right),\ldots \ldots ,\left(x_{N},y_{N}\right)\right\}$, Number of trees: T, The number of features randomly selected by each node K, The number of split points randomly selected for each feature S 2. For each tree t=1,2,……,T - subset is randomly selected from D - Construct decision tree recursively: i. At each node, K features are randomly selected ii. For each selected feature, S split points are randomly selected iii. And randomly choose one of them as the split point iv. The data set is divided into left and right child nodes v. based on the selected split point - Repeat the process until the stop condition is met - Prediction - The predicted results of all trees are averaged to obtain the final regression prediction value 3. Output Surface roughness prediction value

## References

[1] Xie S., He Z., Loh Y.M., Yang Y., Liu K., Liu C., Cheung C.F., Yu N., Wang C. (2024). A novel interpretable predictive model based on ensemble learning and differential evolution algorithm for surface roughness prediction in abrasive water jet polishing. J. Intell. Manuf..

[2] Tian W., Zhang J., Zhao F., Feng X., Mei X., Chen G., Wang H. (2024). Interpolation-based virtual sample generation for surface roughness prediction. J. Intell. Manuf..

[3] Yang HGZheng H., Zhang T.H. (2024). A review of artificial intelligent methods for machined surface roughness prediction. Tribol. Int..

[4] Yeganefar A., Niknam S.A., Asadi R. (2019). The use of support vector machine, neural network, and regression analysis to predict and optimize surface roughness and cutting forces in milling. Int. J. Adv. Manuf. Technol..

[5] Wang J., Wu X., Huang Q., Mu Q., Yang W., Yang H., Li Z. (2025). Surface roughness prediction based on fusion of dynamic-static data. Measurement.

[6] Çaydas U., Ekici S. (2012). Support vector machines models for surface roughness prediction in CNC turning of AISI 304 austenitic stainless steel. J. Intell. Manuf..

[7] Pimenov D.Y., Bustillo A., Mikolajczyk T. (2018). Artificial intelligence for automatic prediction of required surface roughness by monitoring wear on face mill teeth. J. Intell. Manuf..

[8] Siyambas Y., Akdulum A. (2024). Prediction of surface roughness using different features in vortex cooled turning process of Ti6Al4V alloy. Measurement.

[9] Zhang Y., Xu X.J. (2022). Machine learning surface roughnesses in turning processes of brass metals. Int. J. Adv. Manuf. Technol..

[10] Wang J.J., Ma Y.L., Zhang L.B., Gao R.X., Wu D.Z. (2018). Deep learning for smart manufacturing: Methods and applications. J. Manuf. Syst..

[11] Xiao Y.Z., Zheng S., Shi J.C., Du X.D., Hong J. (2023). Knowledge graph-based manufacturing process planning: A state-of-the-art review. J. Manuf. Syst..

[12] Pan Y.A., Kang R.K., Dong Z.G., Du W.H., Yin S., Bao Y. (2022). On-line prediction of ultrasonic elliptical vibration cutting surface roughness of tungsten heavy alloy based on deep learning. J. Intell. Manuf..

[13] Guo W.C., Wu C.J., Ding Z.S., Zhou Q.Z. (2021). Prediction of surface roughness based on a hybrid feature selection method and long short-term memory network in grinding. Int. J. Adv. Manuf. Technol..

[14] Lin W.J., Lo S.H., Young H.T., Hung C.L. (2019). Evaluation of Deep Learning Neural Networks for Surface Roughness Prediction Using Vibration Signal Analysis. Appl. Sci..

[15] Cao C., Zhao Y., Song Z., Dai D., Liu Q., Zhang X., Meng J., Gao Y., Zhang H., Liu G. (2022). Prediction and Optimization of Surface Roughness for Laser-Assisted Machining SiC Ceramics Based on Improved Support Vector Regression. Micromachines.

[16] Li W., Zhang L.C., Chen X.P., Wu C.H., Cui Z.X., Niu C. (2021). Predicting the evolution of sheet metal surface scratching by the technique of artificial intelligence. Int. J. Adv. Manuf. Technol..

[17] Boga C., Koroglu T. (2021). Proper estimation of surface roughness using hybrid intelligence based on artificial neural network and genetic algorithm. J. Manuf. Process..

[18] Zhou T., He L., Wu J.X., Du F.L., Zou Z.F. (2019). Prediction of Surface Roughness of 304 Stainless Steel and Multi-Objective Optimization of Cutting Parameters Based on GA-GBRT. Appl. Appl. Sci..

[19] Sheng Y., Zhang G., Zhang Y., Luo M., Pang Y., Wang Q. (2024). A multimodal data sensing and feature learning-based self-adaptive hybrid approach for machining quality prediction. Adv. Eng. Inform..

[20] Jiang Y.M., Ma X.Y., Li X. (2025). Towards virtual sample generation with various data conditions: A comprehensive review. Inf. Fusion..

[21] Wen L., Li X.Y., Gao L., Yi W.C. (2016). Surface roughness prediction in end milling by using predicted poInt. oriented local linear estimation method. Int. J. Adv. Manuf. Technol..

[22] Wang Y.Q., Niu M.M., Liu K., Shen M.R., Qin B., Wang H.H. (2023). A Novel Data Augmentation Method Based on CoralGAN for Prediction of Part Surface Roughness. IEEE Trans. Neural. Netw. Learn. Syst..

[23] Cooper C., Zhang J.J., Guo Y.B., Gao R.X. (2023). Surface roughness prediction through GAN-synthesized power signal as a process signature*. J. Manuf. Syst..

[24] Wang Y., He Z., Xie S., Wang R., Zhang Z., Liu S., Shang S., Zheng P., Wang C. (2024). Explainable prediction of surface roughness in multi-jet polishing based on ensemble regression and differential evolution method. Expert Syst. Appl..

[25] Ruan P.H., Saxena D., Cao J.N., Liu X.Y., Wang R.X., Cheung C.F. (2025). NASPrecision: Neural Architecture Search-Driven Multi-Stage Learning for surface roughness prediction in ultra-precision machining. Expert Syst. Appl..

[26] Chen N., Zhao S., Gao Z., Wang D., Liu P., Oeser M., Hou Y., Wang L. (2022). Virtual mix design: Prediction of compressive strength of concrete with industrial wastes using deep data augmentation. Constr. Build. Mater..

[27] Brock A., Donahue J., Simonyan K. (2018). Large scale GAN training for high fidelity natural image synthesis. arXiv.

[28] Yu A., Pan Y., Wan F., Sun G., Zhang J., Lu X. (2024). Rapid accomplishment of cost-effective and macro-defect-free LPBF-processed Ti parts based on deep data augmentation. J. Manuf. Process..

[29] Yu H., Fan X., Wang G., Xie Y. (2023). VSG 3 A 2: A Genetic Algorithm-Based Virtual Sample Generation Approach Using Information Gain and Acceptance-Rejection Sampling. IEEE Trans. Evol. Comput..

[30] Kahan W. (1996). IEEE standard 754 for binary floating-point arithmetic. Lect. Notes Status IEEE.

[31] Geurts P., Ernst D., Wehenkel L. (2006). Extremely randomized trees. Mach. Learn..

[32] Franco L.A., Sinatora A. (2015). 3D surface parameters (ISO 25178-2): Actual meaning of Spk and its relationship to Vmp. Precis. Eng..

[33] Wang C., Loh Y.M., Cheung C.F., Liang X., Zhang Z., Ho L.T. (2023). Post processing of additively manufactured 316L stainless steel by multi-jet polishing method. J. Mater. Res. Technol..

[34] Cortes C. (1995). Support-Vector Networks. Mach. Learn..

[35] Breiman L. (2001). Random forests. Mach. Learn..

[36] Friedman J.H. (2001). Greedy function approximation: A gradient boosting machine. Ann. Stat..

[37] Chen T., Guestrin C. Xgboost: A scalable tree boosting system. Proceedings of the 22nd Acm Sigkdd International Conference on Knowledge Discovery and Data Mining.

[38] Rumelhart D.E., Hinton G.E., Williams R.J. (1986). Learning representations by back-propagating errors. Nature.

[39] Quinlan J.R. (1986). Induction of decision trees. Mach. Learn..

[40] Cover T., Hart P. (1967). Nearest neighbor pattern classification. IEEE Trans. Inf. Theory.

[41] Hancock J.T., Khoshgoftaar T.M. (2020). CatBoost for big data: An interdisciplinary review. J. Big Data.

[42] Goodfellow I., Pouget-Abadie J., Mirza M., Xu B., Warde-Farley D., Ozair S., Courville A., Bengio Y. (2020). Generative adversarial networks. Commun. ACM.

[43] Doersch C. (2016). Tutorial on variational autoencoders. arXiv.

[44] Shapley L.S., Kuhn H., Tucker A. (1953). A value for n-person games. Contributions to the Theory of Games II.

[45] Lundberg S.M., Lee S.-I. (2017). A unified approach to interpreting model predictions. Adv. Neural Inf. Process. Syst..

